# Prevalence of asthma symptoms and associated factors in adolescents and adults in southern Brazil: A *Global Asthma Network* Phase I study^☆^

**DOI:** 10.1016/j.waojou.2021.100529

**Published:** 2021-03-17

**Authors:** Marilyn Urrutia-Pereira, Herberto Chong-Neto, Lucas Pitrez Mocellin, Philippa Ellwood, Luis Garcia-Marcos, Laura Simon, Pietro Rinelli, Dirceu Solé

**Affiliations:** a Department of Pediatrics, Federal University of Pampa (Unipampa), Brazil; b Department of Pediatrics, Federal University of Paraná, Brazil; c Department of Collective Health, Faculty of Medicine, Federal University of Pampa, Brazil; d Department of Paediatrics: Child and Youth Health, The University of Auckland, New Zealand; e Pediatric Allergy and Pulmonology Units, Arrixaca University Children's Hospital, University of Murcia, IMIB Bio-health Research Institute of Murcia (IMIB), ARADyAL Network, Spain; f Division of Allergy, Clinical Immunology and Rheumatology, Department of Pediatrics, Federal University of São Paulo, Brazil

**Keywords:** Adolescents, Adults, Asthma, Epidemiology, Risk factors, GAN, Global Asthma Network, HDI, Human Development Index, ISAAC, International Study of Asthma and Allergies in Childhood

## Abstract

**Background:**

The Global Asthma Network (GAN) aims to find out the current status of the prevalence and severity of asthma, rhinitis, and eczema using global surveillance to achieve worldwide recognition and improve the management of asthma, especially in low- and middle-income countries. The aim of this study was to verify the associated factors for asthma in adolescents and their respective parents/caregivers.

**Methods:**

Adolescents (13–14 years old; n = 1058) and their respective parents/caregivers (mean age = 42.1 years, n = 896) living in the town of Uruguaiana, Southern Brazil fulfilled the standardized questionnaire.

**Results:**

Although the prevalence of wheezing in the past 12 months was higher among adults than adolescents (18.4% vs. 15.8%, respectively), adolescents showed more severe wheezing and worse control over the disease revealed by higher consumption of short-acting beta-2 agonists; going to the emergency room; hospitalization in the last year and dry night cough. Smoking and paracetamol use were associated with risk for developing asthma symptoms and consuming seafood/fish was protective. For the adults smoking (10 or more cigarettes/day) and exposure to mould in the house were associated with risk for asthma symptoms.

**Conclusions:**

Adolescents have a high prevalence of asthma symptoms and few have an action plan. Adults do not have their disease under control and they use more relief than preventive medication. Differences in associated factors could determine the outcomes in asthma control among adolescents and their parents.

## Introduction

Asthma is a highly prevalent, chronic respiratory disease and constitutes a significant public health problem around the world. It has high direct and indirect costs and significantly impairs the quality of life of patients and their families.^1^

The Global Asthma Network (GAN) was formed in 2012 and followed on from the International Study of Asthma and Allergies in Childhood (ISAAC). GAN aims to conduct asthma surveillance around the world to achieve global recognition and improve asthma management, especially in low- and middle-income countries.^2^ Such goals will be achieved through improved monitoring, research, training, and access to effective asthma care, including quality assured essential medicines.^2^ A standardized questionnaire based on ISAAC Phase Three^3^ allowed us to obtain the prevalence of asthma in adolescents and their parents/caregivers, making it easier to compare between the different centers (local and from other countries) in both developed and developing countries.^2^

GAN Phase I assessed the symptom prevalence and severity of asthma in adolescents and their parents/caregivers living in the city of Uruguaiana, Southern Brazil. We also evaluated risk factors, asthma management, and other factors in both populations.

## Method

### Study design

This was a cross-sectional study performed by Uruguaiana GAN Phase I center involving 13–14 years olds (adolescents) and their parents/caregivers (adults) residing in the municipality, following the standard GAN protocol.^2^

Data were collected from randomly selected public schools in the city within the geographic radius of the sampling frame, from October 2016 to November 2018. One thousand two hundred (1200) adolescents from 17 schools were invited to participate^4^ and 1058 adolescents participated. Questionnaires that were completely blank or contained only demographic data were excluded. The adolescents completed the questionnaires in their classroom under the supervision of trained volunteer fellows.

The adolescents included in the study (n = 1058) took an adult questionnaire home for adult completion (25–75 years, mean = 42.1 years; standard deviation = 8.8 years) and returned within one week. There were 920 questionnaires returned, and 896 of them were filled out correctly.^5^

The Ethics Committee of the Federal University do Pampa (UNIPAMPA), CASE No: 62658216.2000.5323, approved this study. The Terms of Consent and Free and Informed Consent were obtained from the adolescents and their parents/caregivers, respectively.

### Global Asthma Network questionnaires

Standardised GAN questionnaires for (adolescents and adults),^4^^,^^5^ were translated into Portuguese, following the ISAAC protocol for translation, back translation to English, and comparison.^6^ Demographic questions on age, sex, date of birth, school, interview date, and ethnicity were asked. The height and weight, of the adolescents was measured at school by the fieldworkers and participants (adolescents and adults) completed questions on episodes of wheezing, coughing, shortness of breath, asthma diagnosis, rhinitis, eczema, risk factors, and asthma management.^7^ Each questionnaire had a unique code according to the number of the center, school, and the participant to guarantee confidentiality of the participant and to enable the questionnaires to be linked between adults (parents/caregivers) and adolescents.^2^

Affirmative responses to “*have you had wheezing or whistling in the chest in the past* 12 months” identified adolescents and adults as having asthma symptoms.

Severe asthma was identified, both adolescents and adults, using the following criteria: affirmative answer to “*wheeze or whistling in your chest at any time in the past* 12 months” and affirmative answer to at least one of the following criteria: “*more than 12 attacks of wheeze in the past* 12 months” or “*woken one or more nights per week in the past* 12 months” or “*wheezing severe enough to limit speech to only one or two words at a time between breaths*”.

### Sample size

As per ISAAC, a large sample size of between 1000 and 3000 participants is sought, because of the number of hypotheses being tested and a high response rate is also sought as it is a concern that absent school pupils may be away from school due to symptoms of asthma, rhinitis, or eczema.^8^ This is similar to that used in a study carried out in the same municipality between 2005-2006.^8^

### Data collection and analysis

Data was double entered into an Excel database. After comparing the 2 entries and verifying there were no inconsistencies, the data and a centre report which detailed how the methodology was undertaken, were forwarded to global GAN Center in Auckland, New Zealand,^2^ to have initial checks done. These were then sent to the GAN Data Centre in Spain for a comprehensive data check to be undertaken.

Categorical variables were presented in frequency and proportion, and continuous variables as mean and standard deviation. The factors associated with wheezing in the past 12 months were studied using bivariate and multivariate analysis. Factors related to number of older siblings, practice of exercise, and environmental exposure were introduced into the multivariate analysis using p values less than 0.2, from the bivariate analysis, according to the binary logistic regression analysis method. P values less than 0.05 were considered statistically significant.

## Results

The response rate of the adolescents was 88.2%, and for the adults it was 84.6%. There were more male participants among the adolescents (55.1%), which did not occur among parents/caregivers (3.3%). Table 1 shows the participants' affirmative responses for asthma symptoms and treatments. The prevalence of wheezing in the past 12 months was significantly higher among adults as compared to adolescents (18.4% *vs.* 15.8%, respectively), as well as the prevalence of waking up at night due wheezing (9.9 % × 7.6 %, respectively). The prevalence of having had 4 or more wheezing episodes or severe wheezing limiting speech was more frequent among the adolescents as compared to adults (15.4% *vs* 7.4% and/or 4.4% *vs* 2.5%, respectively). As well as visiting the emergency department (15.3% *vs* 1.0%, respectively) and being hospitalized due to wheezing in the past year (15.2% *vs* 0.2%, respectively). Despite that, having a physician diagnosis of asthma and a written treatment plan was lower for both groups. For the adolescents, 7% and 4%, respectively, and for adults, 2.3% and 1.3%, respectively (Table 1).

**Table 1:** Distribution of adolescents and parents/guardians (adults) according to affirmative answers to the Global Asthma Network (asthma) questionnaire – comparative analysis.

Question	Adolescents 1058 (%)	Adults 896 (%)
Wheezing ever	349 (32.9)∗	165 (18.4)
Wheezing past 12 months	167 (15.8)	165 (18.4)∗
Wake up at night due to wheezing	80 (7.6)	89 (9.9)∗
Four or more wheezing episodes	163 (15.4)∗	66 (7.4)
Dry night time cough	109 (10.3)∗	36 (4.0)
Severe wheezing limiting speech	47 (4.4)∗	22 (2.5)
Emergency room visits	162 (15.3)∗	9 (1.0)
At least one hospitalization due to wheezing in the past year	161 (15.2)∗	2 (0.2)
Physician diagnosed asthma	74 (7.0)∗	21 (2.3)
Severe asthma	66 (6.2)	69 (7.6)∗
To have a written asthma plan of treatment	44 (4.1)∗	12 (1.3)
Inhaled short-acting beta 2 agonist	63 (6.0)∗	17 (1.9)
Inhaled corticosteroids	33 (3.4)∗	16 (1.8)
Missing school and/or work days	70 (6.6)∗	15 (1.7)

Chi-square: ∗p < 0.001 – significantly higher than the other.

Adolescents showed a higher frequency of inability to control self-reported asthma. In the past year, 97.6% manifested 4 or more wheezing episodes, 97.0% sought emergency services because of acute attacks, 96.4% were hospitalized at least once, and 65.2% complained of dry night coughing (Table 1). Despite this, only 44.3% of these adolescents reported having medically diagnosed asthma, 26.3% had a treatment plan, 19.7% were treated with inhaled corticosteroids, and 37.7% used a short-acting beta-2 agonist agent to relieve symptoms as a one-time medication (Table 1). Despite lower proportions of patients being treated, asthma symptoms among adults appeared to be milder (Table 1).

Table 2 shows factors associated with wheezing in the past year for adolescents obtained by logistic regression among the variables identified by the multivariate analysis. The consumption of seafood/fish one or more times a week was protective against developing wheezing for adolescents. Use of paracetamol and exposure to tobacco smoking were identified as risk factors for developing wheezing (Table 2).

**Table 2:** Factors associated with wheezing in the past year in adolescents living in Uruguaiana, Brazil.

Variable	Univariate	Multivariate
		N = 206 LR = 118.70
	OR (95% CI)	aOR (95% CI)
**Number of older siblings**		
One	1.20 (0.73–1.98)	1.28 (0.56–2.94)
Two or more	1.42 (0.87–2.29)	1.33 (0.59–3.02)
**Practice of exercises in a week**		
One or two times	1.39 (0.88–2.20)	1.59 (0.72–3.49)
Three or more times	1.58 (0.92–2.72)	1.75 (0.69–4.44)
**Smoking in the past**		
	1.39 (0.55–3.50)	4.97 (1.01–24.46)∗
**Use of paracetamol**		
Once a year	1.29 (0.73–2.29)	1.52 (0.58–4.01)
Once a month	2.86 (1.62–5.04)∗	4.67 (1.79–12.19)∗
**Seafood consumption**		
One or more times a week	0.29 (0.15–0.57)∗	0.29 (0.10–0.79)∗
**Fruit consumption**		
One or two times a week	1.37 (0.70–2.69)	1.16 (0.38–3.62)
Every day	1.09 (0.55–2.15)	0.76 (0.22–2.57)
**Raw vegetables consumption**		
One or two times a week	0.96 (0.57–1.63)	0.94 (0.43–2.05)
Every day	0.81 (0.45–1.45)	1.17 (0.46–2.98)
**Margarine consumption**		
One or two times a week	1.24 (0.69–2.23)	1.69 (0.69–4.13)
Every day	1.33 (0.78–2.26)	1.18 (0.49–2.83)
**Butter consumption**		
One or two times a week	0.79 (0.45–1.41)	0.49 (0.21–1.17)
Every day	0.84 (0.50–1.41)	0.75 (0.32–1.80)
**Milk consumption**		
One or two times a week	1.24 (0.68–2.28)	0.72 (0.26–1.94)
Every day	1.15 (0.66–2.01)	0.80 (0.32–1.98)
**Sugar consumption**		
One or two times a week	1.03 (0.48–2.22)	1.43 (0.42–4.87)
Every day	1.57 (0.76–3.24)	1.84 (0.57–5.87)
**Pulses consumption**		
One or two times a week	1.19 (0.54–2.62)	2.08 (0.63–6.90)
Every day	0.81 (0.36–1.81)	0.69 (0.19–2.48)
**Olive oil consumption**		
One or two times a week	1.31 (0.63–2.74)	0.78 (0.23–2.64)
Every day	1.32 (0.68–2.57)	0.98 (0.34–2.78)
**Fast-food consumption**		
One or two times a week	1.09 (0.67–1.77)	1.15 (0.56–2.39)
Every day	0.76 (0.31–1.87)	0.41 (0.09–1.81)

N: number of subjects included in analysis; LR: likelihood ratio; OR: Odds ratio; aOR: adjusted Odds ratio; 95%CI: 95% confidence interval; ∗significant values (p < 0.05).

Table 3 shows the variables possibly associated with asthma prevalence among adults. We verified that active smoking with consumption of 10 or more cigarettes/day, as well as being exposed to mould in the house were identified as risk factors for developing asthma and consumption of diary products and olive oil were identified as protective factors for developing asthma (Table 3).

**Table 3:** Factors associated with wheezing in the past year in adults living in Uruguaiana, Brazil.

Variable	Univariate	Multivariate
		N = 330 LR = 125.70
	OR (95% CI)	aOR (95% CI)
Low education	1.67 (1.14–2.43)∗	0.85 (0.34–2.16)
**Smoking status**		
Up to 9 cigarettes a day	1.47 (0.84–2.57)	0.92 (0.36–2.36)
10 or more cigarettes a day	2.74 (1.59–4.73)∗	4.21 (1.01–17.57)∗
**Mould in the house**		
Larger than a postcard	1.36 (1.08–2.47)∗	2.08 (0.93–4.67)
Smaller than a postcard	1.92 (1.27–2.91)∗	2.91 (1.21–7.04)∗
**Use of cooking materials considered a risk for wheezing**^**1**^		
	2.98 (1.31–6.76)∗	3.18 (0.40–25.50)
**Seafood consumption**		
Every day	0.41 (0.09–1.77)	0.54 (0.04–7.80)
One or two times a week	1.07 (0.64–1.78)	1.83 (0.83–4.04)
**Fruit consumption**		
Every day	1.11 (0.46–2.66)	3.82 (0.58–25.03)
One or two times a week	1.04 (0.43–2.51)	2.78 (0.45–17.15)
**Raw vegetables consumption**		
Every day	0.66 (0.35–1.25)	0.52 (0.20–1.34)
One or two times a week	0.82 (0.44–1.54)	0.59 (0.24–1.45)
**Margarine consumption**		
Every day	1.75 (0.99–3.13)	2.03 (0.93–4.43)
One or two times a week	1.54 (0.80–2.98)	1.27 (0.47–3.41)
**Butter consumption**		
Every day	1.07 (0.64–1.81)	1.02 (0.44–2.34)
One or two times a week	1.11 (0.62–2.00)	0.93 (0.39–2.23)
**Milk consumption**		
Every day	1.33 (0.72–2.48)	2.03 (0.79–5.23)
One or two times a week	1.24 (0.67–2.32)	1.62 (0.62–4.25)
**Sugar consumption**		
Every day	2.69 (1.04–6.98)∗	3.10 (0.75–12.93)
One or two times a week	2.12 (0.75–5.94)	2.68 (0.55–12.99)
**Pulses consumption**		
Every day	0.57 (0.23–1.43)	0.13 (0.04–0.50)∗
One or two times a week	0.70 (0.28–1.74)	0.35 (0.09–1.28)
**Olive oil consumption**		
Every day	0.77 (0.30–1.97)	0.30 (0.11–0.79)∗
One or two times a week	0.36 (0.10–1.30)	0.11 (0.02–0.75)∗
**Fast-food consumption**		
Every day	0.97 (0.40–2.36)	1.06 (0.32–3.47)
One or two times a week	1.16 (0.70–1.92)	1.19 (0.56–2.54)

N: number of subjects included in analysis; LR: likelihood ratio; OR: Odds ratio; aOR: adjusted Odds ratio; 95%CI: 95% confidence interval; ^1^list of materials considered in the variable – coal, wood, straw, manure or agricultural crop residues. ∗significant values (p < 0.05).

## Discussion

The Uruguaiana GAN center was the first Brazilian center^9^ to complete collecting data from adolescents and parents/caregivers. Unlike the ISAAC Phase Two study,^10^ conducted in the same center which evaluated 10 year old children, this study had as a complicating factor which was the need for parent/caregiver participation, answering its own questionnaire, because sometimes they lacked the motivation to participate.

The prevalence of asthma in adolescents in this study was lower than those observed by the ISAAC Phase II study in the same center 15 years ago (25.6%)^10^ and those most recently obtained by Brazil's National School Health Survey (PeNSE 2012; 23.2%).^11^ Although carried out at different times, the first 2 studies used the question, “Have you had whistling or wheezing in the chest in the past 12 months”^10^ as an asthma-identifying question and the other question was, “Have you ever had asthma” (medical diagnosis of asthma).^11^ The same occurred concerning the prevalence of severe asthma, which was lower in the current study (6.2% *vs* 7.6%, respectively).^10^

It is worth mentioning that during the interval between ISAAC Phase Two and GAN Phase I, there were improvements in the town's basic sanitation network. This was improved from 16% to 81.2%^12^ and was corroborated by the increase in the Human Development Index (HDI) from 0.523 to 0.744.^13^ Also, the Children's Asthma Prevention Program was created by decree-law. It started to assist children and adolescents with asthma in the municipality referred by primary care and pediatricians and to receive asthma specific treatment.^14^

Population-based studies that have assessed the prevalence of self-reported asthma in adults (≥18 years) are scarce. According to Brazil's National Household Survey (PNAD) in 2003 and 2008, the prevalence of medical diagnosis of asthma was 3.6% and 3.7%, respectively.^15^ Data from Brazil's 2013 National Health Survey (PNS) indicate that the prevalence of asthma in the adult population was 4.4% and was higher among women.^16^ On the other hand, secondary analysis of data from the Cross-Sectional World Health Survey carried out in 70 countries among individuals from 18 to 45 years old^17^ showed that the prevalence of medically diagnosed asthma was 4.3%.^18^ In our study, despite a report of a high frequency of asthma symptoms, only 2.2% of adults reported having a medical diagnosis of asthma, characterizing an underdiagnosis or memory bias similar to what was observed by others.^18^

In the comparative analysis between adolescents and adults, even though adults reported a higher prevalence of wheezing and waking up at night in the past year, adolescents showed a higher frequency of inability to control self-reported asthma.

Adolescents and adults with asthma manifested nocturnal symptoms characterized by a dry cough and waking up at night, certainly compromising their sleep, as previously documented by the authors,^19^ symptoms often underestimated by health care providers due to limited knowledge and inadequate research.^19^^,^^20^ Higher frequency of missing school and/or work days were reported among adolescents compared to adults, which could be due to restless sleep, creating more fatigue, changes in behavior, mood, cognitive functioning, and school performance, as mentioned by other authors in previous studies.^19^^,^^21^

The low prevalence of affirmative responses to the questions, “H*ave you ever had asthma*” and “W*as your asthma confirmed by a doctor?*” in the studied population could be related to denial or less awareness that wheezing is an asthmatic symptom, even in those with frequent wheezing, as identified by a GAN Phase I study in Bangkok.^22^ Despite this, even with the improvement in the HDI, it is not mandatory that they improve adequate healthcare for asthma (access to specialized treatment and medicines) and asthma symptom prevalence could begin a downward spiral and become out of control.^23^ Parents/caregivers may have perceptions or misconceptions about asthma that could lead to ineffective management,^24^^,^^25^ often identified by more frequent visits to the emergency room and even hospitalizations.^26^ Most likely, they are progressive signs of the disease and its lack of control.

However, another issue is that even though asthma-controlling medication is available free of charge throughout the country,^27^ instituting a long-term treatment for controlling asthma symptoms to avoid severe attacks and seek medical assistance is difficult. The participants in the study were rarely taking these medications. This observation may be attributed to the lack of medically diagnosed asthma, lack of adequate education on the disease, or even denial by patients and their caregivers.^1^^,^^23^^,^^24^ We must also emphasize that patients and caregivers tend to overestimate the level of asthma control. They either underestimate its severity or assume that better control is not possible.^28^ The notion of this limited possibility of control stems from the fear, that many patients and caregivers have, of the side effects of inhaled corticosteroids. This corticosteroid phobia (corticophobia) may determine changes in the treatment regimen, or discontinue its use altogether.^29^ Another factor that contributes to inadequate asthma control in adolescents is that few of them adhere to treatment.^30^

The standardised GAN Phase I questionnaire also allowed us to assess the eating habits of the adolescents and adults individuals. This evaluation showed that adolescents consumed fruits, cereals, olive oil, milk, and nuts, in addition to fast food and soft drinks more frequently than adults (at least once a week). Adults consumed more seafood/fish (data not shown). Despite this, consuming seafood was only shown as a protective association against developing asthma among adolescents and consuming olive oil or pulses consumption only among adults (Table 2, Table 3).

Fruits and vegetables have been extensively studied as potent sources of antioxidants.^31^ A worldwide international study documented that frequent consumption of fruits and vegetables is protective against developing current and severe asthma in adolescents.^32^ Although the Mediterranean diet (rich in fish, fruits, and vegetables) has been identified as a healthy eating pattern that can reduce the risk of asthma,^33^ more studies are needed to support the evidence that could lead to considering it a more accurate dietary recommendation.^34^^,^^35^

Consuming fast food has been linked to asthma risk.^32^ In general, it is a food rich in industrially hydrogenated vegetable fats, such as margarine, which are food sources of trans fats (trans unsaturated fatty acids), and studies show an association between its intake and asthma.^31^ Despite this evidence, fast food consumption, in this study, was not associated with the development of asthma in both the adolescents and adults.

Environmental exposure to specific contaminants has been identified as responsible for the development and triggering of asthmatic symptoms among susceptible individuals.^36^ Among adults, exposure to mould in the living room, and active smoking were identified as being associated with increased asthma symptoms (Table 3). Although the adult population consists of more than 96% women, using different sources for cooking was not identified as risk factors for developing asthma.^37^

Our analysis also showed that paracetamol use (once a month) in the past year was a risk factor for asthma among our adolescents. Shaheen et a,^38^ were the first to demonstrate a considerable increase in the risk of asthma symptoms with the highest frequency of paracetamol use. Similarly, a Polish study documented a positive correlation between paracetamol use in the past 12 months and a dose-dependent paracetamol risk of developing symptoms of asthma, rhinitis, and eczema.^39^

One of the strengths in this study is the standardized methods (written questionnaire), which makes it possible to obtain local epidemiological data that allows comparison with other centers in Brazil as well as between different countries. It allowed us to see a reduction in asthma prevalence in the city of Uruguaiana; however, there is still a long way to go.

It is reasonable to consider that answers on international questionnaires, even when validated in several languages, can be influenced by the level of training, education, maturity, understanding, and responsibility of the adolescents that are interviewed. Other important points of concern are the development of the area where the study is carried out and the level of respondents' knowledge of each disease and its symptoms. Parents of adolescents at a social and clinical risk may not accurately report the status of their children. This problem can worsen depending on the socioeconomic level of the family and their education about the diseases and their complications. We emphasize that, although the study found several associations with a higher risk for the development of asthma, its design does not allow establishing a cause and effect relationship.

In conclusion, the prevalence of asthma symptoms among adolescents living in Uruguaiana, despite having decreased, remains high. Not many adults or adolescents have been medically diagnosed with asthma nor do they have a written action plan, and hardly any of them use preventive medication. Identifying and knowing the risk factors for asthma would be a way forward to help families and healthcare professionals to recommend preventive strategies for asthma and its attacks.

## Funding

No fundings.

## Author's consent for publication

All author's approve the final version and consent for publication.

## Author contributions

MUP – leader of local project, data collection and analysis, text production.

HCN – data collection and analysis, text production.

LPM – data analysis and text production.

PE – International leader project, text production.

LGM - International leader project, text production.

LS - data collection and analysis, text production.

PR - data collection and analysis, text production.

DS - data collection and analysis, text production.

## Availability of data and materials

All data and materials are in possession of the authors and available for conference.

## Ethics approval

The Ethics Committee of the Federal University do Pampa (UNIPAMPA) approved this study, CASE No: 62658216.2000.5323. The Terms of Consent and Free and Informed Consent were obtained from the adolescents and their parents/caregivers, respectively.

## Declaration of competing interest

There are no competing interests.
